# Clinical features of superficial and deep peripapillary microvascular density in healthy myopic eyes

**DOI:** 10.1371/journal.pone.0187160

**Published:** 2017-10-26

**Authors:** Mi Sun Sung, Tae Hee Lee, Hwan Heo, Sang Woo Park

**Affiliations:** 1 Department of Ophthalmology and Research Institute of Medical Sciences, Chonnam National University Medical School and Hospital, Gwangju, South Korea; 2 Center for Creative Biomedical Scientists, Chonnam National University, Gwangju, South Korea; The University of Melbourne, AUSTRALIA

## Abstract

**Purpose:**

To evaluate the clinical features of peripapillary microvasculature in myopic eyes and investigate the association between the superficial and deep peripapillary microvascular density and the myopic optic disc characteristics.

**Materials and methods:**

This cross-sectional study included one hundred and fifty healthy myopic eyes with β-peripapillary atrophy (β-PPA). Ovality index, degree of optic disc rotation, and the area of β-PPA were measured. Superficial and deep peripapillary microvascular density was measured using optical coherence tomography angiography. Logistic regression analysis was performed to look for the factors associated with peripapillary microvascular reduction.

**Results:**

The mean superficial peripapillary microvascular density was 62.14 ± 5.47%; 33 (22.0%) participants were found to have decreased microvascular density. Increased axial length (p < 0.001) and decreased average peripapillary retinal nerve fiber layer thickness (p = 0.027) were associated with the superficial peripapillary microvascular reduction. The mean deep peripapillary microvascular density was 73.76 ± 4.02%; 26 (17.33%) participants were found to have decreased microvascular density. Larger ovality index (p = 0.028) and more inferiorly rotated optic disc (p = 0.021) were associated with the deep peripapillary microvascular reduction.

**Conclusions:**

Axial elongation was significantly associated with microvascular reduction in the superficial peripapillary retina, whereas it was not associated with deep peripapillary microvascular reduction. The deep peripapillary microvascular density was independently associated with myopic optic disc characteristics such as ovality index and optic disc rotation.

## Introduction

Myopic eyes, especially highly myopic eyes, demonstrate various morphologic changes in the optic disc, such as β-peripapillary atrophy (β-PPA), optic disc tilt, and rotation. To investigate the anatomic mechanisms of the increased glaucoma susceptibility in myopic eyes, the above mentioned structural changes have been the focus of several studies [1, 2]. It was reported that the direction of optic disc tilt and rotation were important predictors of visual field (VF) defect location in normal-tension glaucoma. Recently, we found that eyes with inferiorly rotated optic disc show more glaucomatous constitutional anatomical changes, such as large β-PPA and reduced peripapillary retinal nerve fiber layer (pRNFL) and macular ganglion cell-inner plexiform layer (mGCIPL) thickness compared to eyes with superiorly rotated optic disc. One of the hypotheses to support our findings may be the difference in peripapillary microvascular circulation between the two groups, which in turn might affect a circulatory state in the prelaminar and laminar region [3]. However, the association between peripapillary microvascular circulatory state and the morphologic changes of myopic optic disc have not been clearly elucidated yet.

Recent advances in optical coherence tomography (OCT) techniques now provide another means of evaluating the retinal and peripapillary microcirculation with the use of the split-spectrum amplitude-decorrelation angiography (SSADA) algorithm [4, 5]. OCT angiography makes it possible to visualize and quantify perfused vessels of the various vascular layers of retina in a fast, reliable, and non-invasive manner [6]. Earlier studies have shown that the blood flow measurements using OCT angiography are reproducible [5, 7]. In the present study, we hypothesize that morphologic changes in the optic disc might be associated with peripapillary microvasculature in myopic eyes. Therefore, the purpose of this study was to evaluate the clinical features of peripapillary microvasculature in healthy myopic eyes and to investigate the association between the peripapillary microvascular density and the myopic optic disc characteristics.

## Materials and methods

### Subjects

The study protocol adhered to the tenets of the Declaration of Helsinki and was approved by the Institutional Review Board of the Chonnam National University Hospital. The participants were informed about the study objectives and written informed consent was obtained from all participants.

Healthy volunteers were prospectively and consecutively recruited from July to September 2016. All subjects underwent complete ophthalmic examination including measurement of best corrected visual acuity (BCVA), intraocular pressure (IOP) using Goldmann applanation tonometry, manifest refraction, slit-lamp examination, anterior chamber angle examination by gonioscopy, ONH and RNFL examination using color stereoscopic disc photography and red-free RNFL fundus photography, and the Swedish Interactive Threshold Algorithm standard 30–2 perimetry with a Humphrey Field Analyzer (Carl Zeiss Meditec Inc., Dublin, CA, USA). All IOP measurements were made between 4:00 and 6:00 pm. Axial length and central corneal thickness were measured using optical low-coherence reflectometry (Lenstar; Haag-Streit AG, Koeniz, Switzerland). A detailed medical history was also recorded for each subject.

The following inclusion criteria were used: healthy subjects aged between 20 and 40 years, a spherical equivalent (SE) refractive error between –12.0 and –0.5 diopters (D), astigmatism within ± 2 D, BCVA of 20/25 or better, IOP ≤ 21 mmHg, normal anterior chamber angles, non-glaucomatous ONHs on stereoscopic photographs (an intact neuroretinal rim without peripapillary hemorrhage, thinning, or localized pallor), absence of any RNFL abnormalities on red-free fundus photographs, and normal VF results in both eyes. In addition, subjects were required to have β-PPA to be included in the study. The β-PPA was defined as an inner crescent of chorioretinal atrophy with visible sclera and choroidal vessels. Because myopic refractive error can be affected by lenticular changes, and aging may increase the incidence of glaucoma, we excluded subjects older than 40 years. In order to increase the yield of healthy myopic eyes, we also excluded extremely highly myopic eyes with an SE < -12.0 D, as it is known that various pathologic changes on the myopic fundus, such as staphyloma, lacquer cracks, etc., increase in prevalence with increasing myopic refractive error. Patients with suspected congenital disc anomaly were excluded. Patients with a horizontally oval disc and situs inversus of the retinal vessels, suggestive of tilted disc syndrome, were also excluded from the analysis. Patients with family history of glaucoma in a first-degree relative, history of intraocular or refractive surgery, pathologic myopia (patch chorioretinal atrophy, lacquer crack lesions, intrachoroidal cavitations, choroidal neovascularization), other evidence of retinal pathology, or opaque media were excluded.

Eligibility was determined by two glaucoma specialists (M.S.S and S.W.P). Evaluators were masked to all other patient and ocular data, and an eye was excluded from study analyses if a consensus could not be reached. In cases where both eyes of a subject met the inclusion criteria, only one eye was randomly selected for the study.

### Evaluation of optic disc characteristics

Digital retinal photographs were obtained using standard settings with a non-mydriatic retinal camera. Each photograph was exported to a desktop computer as a TIFF image file. Using public-domain Java-based image processing software developed by the National Institutes of Health (ImageJ, Version 1.4.1; Wayne Rasband; National Institutes of Health, Rockville, MD, USA), ovality index, optic disc rotation degree, and areas of the optic disc and β-PPA were measured by two independent examiners (M.S.S and T.H.L). Averaged data were used in the final analysis.

The measurement of ovality index and optic disc rotation has been previously described [1–3, 8]. Briefly, ovality index was defined as the ratio between the longest and shortest diameters of the optic disc. Optic disc rotation was defined as the deviation of the long axis of the optic disc from the reference line, which is 90° from a horizontal line connecting the fovea and the center of the optic disc. The angle between the long axis of the optic disc and the reference line was termed the degree of optic disc rotation. Superior rotation indicated clockwise rotation of the optic disc in the right eye or counter-clockwise rotation of the optic disc in the left eye; inferior rotation indicated counter-clockwise rotation of the optic disc in the right eye or clockwise rotation of the optic disc in the left eye. Superior rotation was presented as a negative value, and inferior rotation was presented as a positive value.

The area of the optic disc and β-PPA was determined on the fundus photograph images as the total number of pixels using ImageJ software in a circumferential pattern. Combined with a fundus camera magnification factor of 1.4×, the total magnification by the camera and ImageJ system was calculated. We converted the area from pixels to mm^2^. The area was corrected using Littmann’s formula for each axial length [9]. The anterior corneal curvature radius was set at 7.8 mm, which is the reported mean for Caucasian and Chinese individuals [10].

### Optical coherence tomography angiography imaging

The AngioVue incorporated in the Avanti spectral domain OCT (RTVue-XR Avanti; Optovue, Fremont, CA, USA) was used for the angiography imaging. To identify perfused vessels, the volumetric scans were processed using the SSADA algorithms. Details about Avanti OCT and the SSADA algorithm have been described by previous studies [4, 5, 7, 11].

The AngioVue disc mode automatically segmented the optic disc and peripapillary region into various layers. For the measurement of superficial peripapillary microvascular density, the radial peripapillary capillary (RPC) segment extending from the internal limiting membrane (ILM) to the RNFL was analyzed. Vessel density is defined as the percentage area occupied by vessels in a particular region. The software automatically fitted an ellipse to the optic disc margin and calculated the average vessel density within and around the optic disc. After reviewing the accuracy of the optic disc margin, the line was adjusted manually. Vessel density within the optic disc were calculated from the nerve head segment of the ONH angiogram and referred to as inside disc vessel density. The nerve head segment is defined as the layer that extends from the ILM to 150μm below the ILM. Average peripapillary vessel density was measured in the region defined as a 0.75-mm-wide elliptical annulus extending from the optic disc boundary on the RPC segment. Whole en face vessel density was measured over the entire 4.5 x 4.5 mm scan area. Among the three vessel density summary parameters, the average peripapillary vessel density of RPC segment was used as the superficial peripapillary microvascular density.

**Fig 1:**
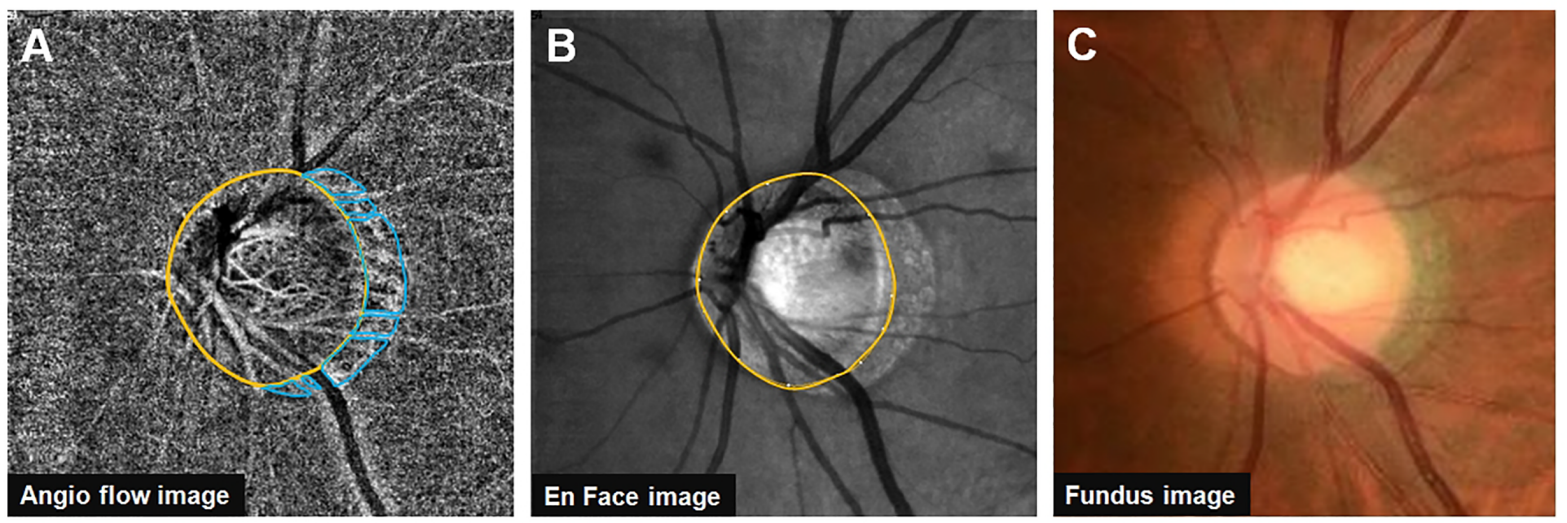
Measurement of deep peripapillary microvascular density in the β-peripapillary atrophy (β-PPA) area. (A) Boundaries of the optic disc, β-PPA, and overlying superficial large vessels delineated. After excluding the area occupied by overlying superficial large vessels, the β-PPA was divided into 7 areas. The choroidal vessel density of each area was automatically measured using the built-in drawing tool of Avanti OCT software. The average choroidal vessel density of 7 areas was calculated and referred to as deep peripapillary microvascular density. (B-C) Corresponding scanning laser ophthalmoscopy image (B) and fundus photography image (C).

For the measurement of deep peripapillary microvascular density, choroidal microvasculature within the β-PPA was evaluated. The software automatically calculated the vessel density of the selected area that was manually traced. After acquiring both angiography and en face image centered on the optic disc at the same position, the boundaries of the optic disc, β-PPA, and superficial large vessels were delineated. To avoid the inaccurate measurement of deep peripapillary microvascular density because of reflectance or shadowing of the overlying superficial large vessels, the area occupied by the superficial large vessels was excluded from the measurements. Average choroidal vessel densities in the remaining β-PPA area were calculated and referred to as the deep peripapillary microvascular density (Fig 1). Measurements were made by two independent examiners (M.S.S and T.H.L) and averaged data were used in the final analysis.

Additionally, all subjects also underwent pRNFL thickness measurement on Avanti spectral domain OCT using the traditional ONH scans. The overall average pRNFL thickness was used in the analysis. A single experienced operator blinded to all the clinical data acquired all OCT scans. ONH angiography and traditional ONH map images of all subjects were reviewed to ensure proper segmentation of OCT scans. Scans with a signal strength index less than 70, segmentation error, or significant motion artifact were excluded.

### Statistical analysis

SPSS version 19.0 (SPSS, Chicago, IL, USA) was used for all statistical analyses. Agreement on several parameters between two observers was assessed using the Bland-Altman method, which plots their means against their differences [12]. The limits of agreement were defined as the mean differences of 2 measurements ± 1.96 standard deviation of the difference. Intraclass correlation coefficients (ICCs) were also calculated. The normality of distribution was verified using the Shapiro-Wilk normality test. Groups were compared using the chi-square, independent *t*, or Mann-Whitney *U* tests as appropriate. Logistic regression analysis was used to investigate baseline factors associated with peripapillary microvascular reduction. First, each variable was analyzed in a univariate model. Next, all variables with a significance level of less than 0.10 were included in the multivariate model. The role of each variable is expressed as odds ratios (OR) and 95% confidence intervals. The relationship between vessel density and significant parameters was additionally examined using scatter plots and linear regression. The coefficient of determination (R^2^) in the linear regression was reported and statistical significance was considered at p < 0.05.

## Results

During the enrollment period, 163 subjects were evaluated. Of these, 2 subjects were excluded because of VF defects or glaucomatous ONH changes, 1 because of other retinal pathologic features, and 10 because of unacceptable OCT image quality. Subsequently, 150 eligible subjects were included in the analyses (S1 Table). The inter-observer agreement, determined using Bland-Altman plots, in the measurements of the ovality index, optic disc rotation, area of the optic disc and β-PPA, and deep peripapillary microvascular density for all subjects showed no systematic differences in measurements. And the ICCs for these measurements were excellent (ICC of ovality index = 0.985, ICC of optic disc rotation degree = 0.974, ICC of disc area = 0.977, ICC of β-PPA area = 0.971, ICC of deep peripapillary microvascular density = 0.965).

The demographic and clinical characteristics of participants are summarized in Table 1. An SE refractive error of less than -6 D (the definition of high myopia) was found in 52 participants. When the subjects were divided into the non-high myopic and high myopic groups, the high myopic group had more inferiorly rotated optic disc (p = 0.042), larger β-PPA (p = 0.010), and thinner average pRNFL thickness (p < 0.001) than did the non-high myopic group. Whole en face vessel density (p < 0.001) and superficial peripapillary microvascular density (p < 0.001) were both significantly lower in highly myopic eyes. When the subjects were divided into the superior and inferior rotation of the optic disc groups, the inferior rotation group had higher IOP (p = 0.009), greater degree of optic disc rotation (p < 0.001), and larger β-PPA (p = 0.002) than did the superior rotation group. Deep peripapillary microvascular density was significantly lower in eyes with inferiorly rotated optic disc (p < 0.001) (Table 1).

**Table 1:** Comparison of subject characteristics according to the myopic degree and optic disc rotation direction.

Variables	Overall (n = 150)	Degree of myopia			Optic disc rotation direction		
		Non-high myopia (n = 98)	High myopia (n = 52)	p value*	Superior (n = 79)	Inferior (n = 71)	p value^†^
**Age (yrs)**	23.39 ± 3.85	23.80 ± 3.66	22.62 ± 4.11	0.074	23.44 ± 3.93	23.32 ± 3.78	0.851
**Sex (male/female)**	99 / 51	64 / 34	35 /17	0.476	55 / 24	44 / 27	0.389
**SE refractive error (D)**	-5.34 ± 2.72	-3.72 ± 1.40	-8.38 ± 1.83	**< 0.001**	-4.96 ± 2.49	-5.76 ± 2.91	0.077
**IOP (mmHg)** ^‡^	14.87 ± 2.44	14.70 ± 2.35	15.17 ± 2.61	0.265	14.38 ± 2.58	15.41 ± 2.17	**0.009**
**Axial length (mm)**	26.05 ± 1.25	25.46 ± 0.98	27.14 ± 0.96	**< 0.001**	25.96 ± 1.23	26.14 ± 1.29	0.379
**Central corneal thickness (μm)**	558.21 ± 35.37	558.34 ± 37.64	557.96 ± 31.01	0.951	555.72 ± 30.52	260.97 ± 40.14	0.373
**Disc area (mm**^**2**^**)**	1.86 ± 0.40	1.88 ± 0.45	1.80 ± 0.25	0.071	1.85 ± 0.33	1.87 ± 0.46	0.818
**Vertical CDR**	0.48 ± 0.15	0.49 ± 0.16	0.45 ± 0.15	0.081	0.45 ± 0.14	0.61 ± 0.17	0.061
**Ovality index**	1.31 ± 0.13	1.31 ± 0.12	1.33 ± 0.14	0.340	1.31 ± 0.13	1.32 ± 0.12	0.609
**Optic disc rotation degree (°)**	1.57 ± 15.60	-0.31 ± 15.07	5.12 ± 16.10	**0.042**^§^	-11.95 ± 4.66	16.62 ± 7.60	**< 0.001**^§^
**Area of β-PPA (mm**^**2**^**)**	0.96 ± 0.42	0.90 ± 0.41	1.08 ± 0.41	**0.010**	0.86 ± 0.37	1.07 ± 0.45	**0.002**
**Average pRNFL thickness (μm)**	98.71 ± 7.97	100.95 ± 6.79	94.48 ± 8.37	**< 0.001**	99.27 ± 7.09	98.08 ± 8.86	0.367
**Whole en face vessel density of RPC segment (%)**	55.16 ± 2.40	55.74 ± 2.24	54.06 ± 2.35	**< 0.001**	55.46 ± 2.08	54.82 ± 2.69	0.100
**Inside disc vessel density (%)**	47.66 ± 8.13	46.85 ± 8.51	49.17 ± 7.21	0.097	48.79 ± 7.99	46.39 ± 8.15	0.073
**Superficial peripapillary microvascular density (%)**	62.14 ± 5.47	63.03 ± 2.56	60.49 ± 3.46	**< 0.001**	62.38 ± 2.90	61.88 ± 3.39	0.336
**Deep peripapillary microvascular density (%)**	73.76 ± 4.02	73.60 ± 4.22	74.05± 3.64	0.525	75.05± 3.71	72.32± 3.89	**< 0.001**

SE = spherical equivalent; D = diopters; IOP = intraocular pressure; CDR = cup-to-disc ratio; PPA = peripapillary atrophy; pRNFL = peripapillary retinal nerve fiber layer; RPC = radial peripapillary capillary.

* Comparisons between the non-high myopia and high myopia groups using an independent-*t* test or chi-square test as appropriate.

^†^ Comparisons between the superior rotation and inferior rotation groups using an independent-*t* test or chi-square test as appropriate.

^‡^ All IOP measurements were made between 4:00 and 6:00 pm.

^§^ The comparison was done with the unsigned value.

In the present study, to find the factors associated with peripapillary microvascular reduction in healthy myopic eyes, we categorized the participants based on the superficial and deep peripapillary microvascular densities. Arbitrarily chosen cut-off values were 60% and 70% for the superficial retina and the choroid, respectively. The cut-off values were based on the lower quartile values of each vessel density parameter (lower quartile values of superficial peripapillary microvascular density, 60.52%; lower quartile values of deep peripapillary microvascular density, 70.71%). Of 150 subjects, 33 (22.0%) subjects showed superficial peripapillary microvascular reduction and 26 (17.33%) subjects showed deep peripapillary microvascular reduction. Seven subjects showed a decrease in both superficial and deep peripapillary microvascular densities. None of the subjects in the present study had a deep microvasculature drop out.

Tables 2 and 3 show the results of logistic regression analyses evaluating the association between various ocular parameters and the peripapillary microvascular density in healthy myopic eyes. In multivariate analysis, longer axial length (OR = 2.756, p < 0.001) and thinner average pRNFL thickness (OR = 0.927, p = 0.027) were statistically significantly associated with superficial peripapillary microvascular reduction. However, deep peripapillary microvascular density showed no significant association with axial length and average pRNFL thickness. Multivariate analysis revealed that larger ovality index (OR = 1.664, p = 0.028) and more inferiorly rotated optic disc (OR = 1.041, p = 0.021) were independently associated with deep peripapillary microvascular reduction. In addition, inside disc vessel density was also identified as a factor related to the deep peripapillary microvascular reduction (OR = 0.919, p = 0.008).

**Table 2:** Factors associated with superficial peripapillary microvascular reduction (< 60%) in healthy myopic eyes.

Variables	Univariate Analysis			Multivariate Analysis*		
	Odds Ratio	95% CI	p value	Odds Ratio	95% CI	p value
**Age, per 1 yr older**	0.919	0.824–1.025	0.130			
**Female gender**	0.665	0.399–1.334	0.204			
**IOP, per 1 mmHg higher**	1.187	1.005–1.400	**0.043**	1.214	0.980–1.504	0.076
**Axial length, per 1mm longer**	3.305	2.069–5.280	**< 0.001**	2.756	1.600–4.749	**< 0.001**
**Central corneal thickness, per 1μm thicker**	0.997	0.986–1.008	0.566			
**Disc area, per 1 mm**^**2**^ **increase**	0.798	0.289–2.199	0.662			
**Vertical CDR, per 0.1 increase**	0.556	0.457–3.521	0.407			
**Ovality index, per 0.1 increase**	1.460	1.064–2.003	**0.019**	1.100	0.725–1.671	0.654
**Rotation degree, per 1° increase**	1.023	0.998–1.049	0.068	1.001	0.968–1.036	0.936
**Area of β-PPA, per 1 mm**^**2**^ **increase**	10.465	3.418–32.037	**< 0.001**	3.636	0.983–13.449	0.053
**Average pRNFL thickness, per 1 μm increase**	0.889	0.839–0.941	**< 0.001**	0.927	0.866–0.992	**0.027**
**Inside disc vessel density, per 1% increase**	1.071	0.881–1.415	0.320			
**Deep peripapillary microvascular density, per 1% increase**	1.008	0.915–1.110	0.872			

CI = confidence interval; IOP = intraocular pressure; CDR = cup-to-disc ratio; PPA = peripapillary atrophy; pRNFL = peripapillary retinal nerve fiber layer; RPC = radial peripapillary capillary.

* Variables with p < 0.10 in the univariate model were entered in a multivariate model.

**Table 3:** Factors associated with deep peripapillary microvascular reduction (< 70%) in healthy myopic eyes.

Variables	Univariate Analysis			Multivariate Analysis*		
	Odds Ratio	95% CI	p value	Odds Ratio	95% CI	p value
**Age, per 1 yr older**	1.072	0.964–1.193	0.200			
**Female gender**	1.034	0.425–2.516	0.942			
**IOP, per 1 mmHg higher**	1.168	0.977–1.398	0.088	1.115	0.894–1.392	0.334
**Axial length, per 1mm longer**	0.966	0.688–1.356	0.842			
**Central corneal thickness, per 1μm thicker**	1.006	0.994–1.018	0.313			
**Disc area, per 1 mm**^**2**^ **increase**	2.534	0.561–5.242	0.210			
**Vertical CDR, per 0.1 increase**	15.35	0.662–32.67	0.312			
**Ovality index, per 0.1 increase**	1.496	1.060–2.111	**0.022**	1.664	1.058–2.618	**0.028**
**Rotation degree, per 1° increase**	1.049	1.019–1.080	**0.001**	1.041	1.006–1.076	**0.021**
**Area of β-PPA, per 1 mm**^**2**^ **increase**	3.937	1.486–10.428	**0.006**	1.653	0.487–5.615	0.420
**Average pRNFL thickness, per 1 μm increase**	0.958	0.907–1.011	0.116			
**Whole en face vessel density of RPC segment, per 1% increase^†^**	0.897	0.758–1.063	0.210			
**Inside disc vessel density, per 1% increase**	0.939	0.893–0.987	**0.013**	0.919	0.863–0.979	**0.008**
**Superficial peripapillary microvascular density, per 1% increase**	0.992	0.867–1.135	0.907			

CI = confidence interval; IOP = intraocular pressure; CDR = cup-to-disc ratio; PPA = peripapillary atrophy; pRNFL = peripapillary retinal nerve fiber layer; RPC = radial peripapillary capillary.

* Variables with p < 0.10 in the univariate model were entered in a multivariate model.

^†^ Because the measurement of whole en face vessel density includes the superficial peripapillary microvascular density, the whole en face vessel density was not incorporated in the multivariate analysis.

**Fig 2:**
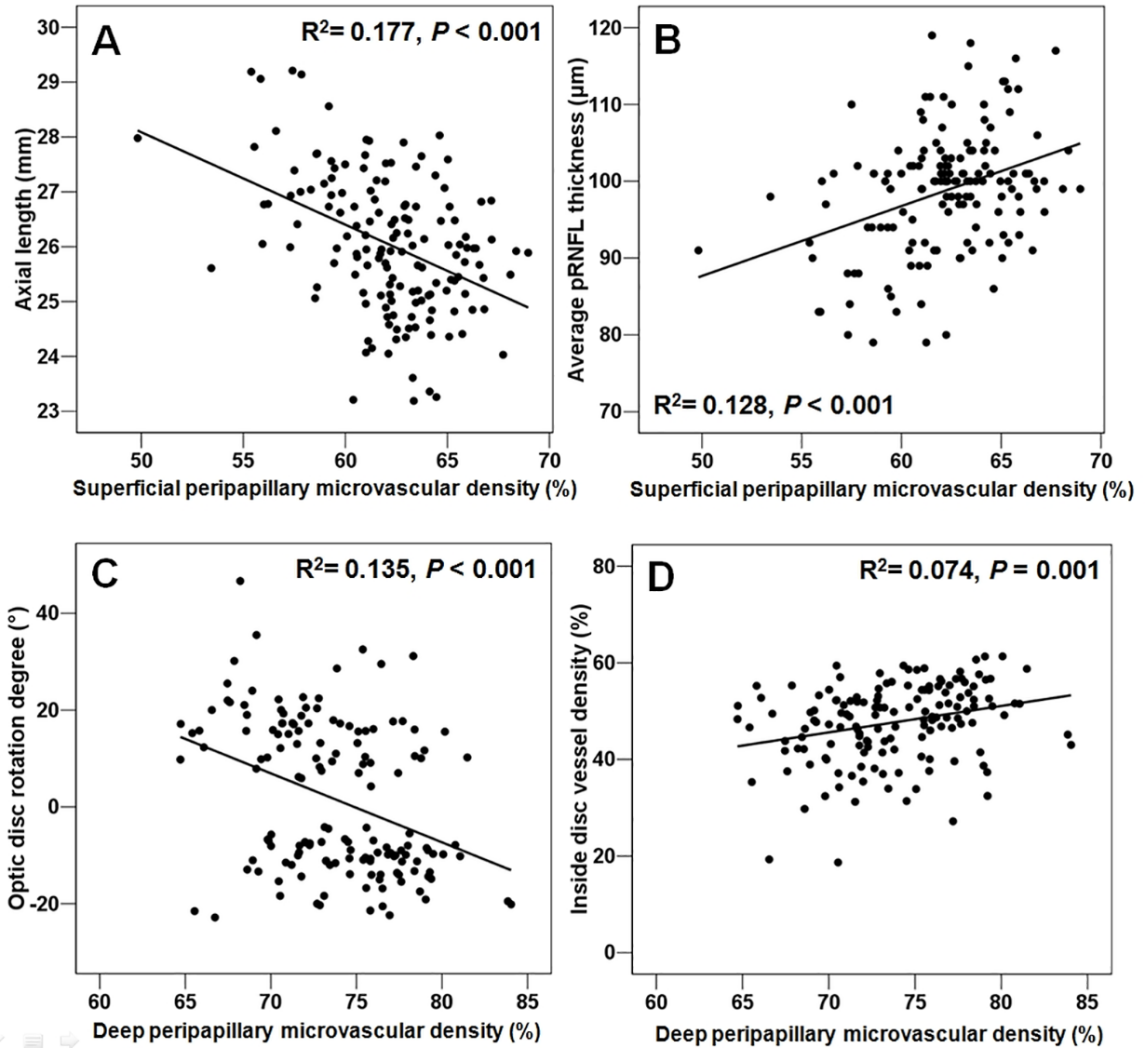
Scatter plots illustrating the linear correlation between peripapillary microvascular density and ocular parameters. (A-B) Relationship between the superficial peripapillary microvascular density and axial length (A) and average peripapillary retinal nerve fiber layer (pRNFL) thickness (B). (C-D) Relationship between the deep peripapillary microvascular density and degree of optic disc rotation (C) and inside disc vessel density (D).

Fig 2 illustrates the relationship between the significantly related factors in logistic regression analysis and the superficial and deep peripapillary microvascular densities. Among the significant factors, ovality index was not found to have a statistically significant linear relationship with deep peripapillary microvascular density (R^2^ = 0.017, p = 0.116).

## Discussion

This study has shown that superficial peripapillary microvascular density measured using an OCT angiography is correlated with axial length and average pRNFL thickness. However, deep peripapillary microvascular density was not found to have a significant association with axial length and average pRNFL thickness. Instead, deep peripapillary microvascular density was affected by myopic optic disc morphology, such as ovality index and optic disc rotation. To our knowledge, this is the first study to evaluate the relationship between optic disc morphology and peripapillary microvasculature in healthy myopic eyes.

RPC, which is the most superficial layer of capillaries lying in the inner part of the RNFL, supplies the retinal ganglion cell axons. A growing body of evidence for the association between RPC loss and RNFL changes in glaucoma has been reported [7, 13, 14]. It has been postulated that decreased density of retinal microvasculature probably represents the closure or degeneration of capillaries that occurs with RNFL loss. In the present study, we also found a significant positive correlation between average pRNFL thickness and superficial peripapillary microvascular density. Thinning of RNFL may affect regional oxygen demand or the need of vascular supply in the peripapillary region, and thereby trigger the retinal vascular adjustment via autoregulatory mechanisms. Xu *et al* [15] have shown that there is a significant reduction in perfused superficial retinal vessel density after hyperoxia, which supports the autoregulatory adaptation of retinal microcirculation.

Retinal perfusion in myopia has been an important issue for several decades because it might provide us a critical clue to understanding the pathophysiology of various myopia-related diseases including glaucoma. Reduced retinal vessel density or blood flow in highly myopic patients has already been reported using different techniques [16–18]. Recently, using OCT angiography, Li *et al* [19] and Yang *et al* [20] have shown that there are retinal microvascular alterations in highly myopic eyes, which correlates with axial elongation. They reported that highly myopic eyes had decreased microvascular density in macular region compared with control groups. In line with their findings, we observed that axial elongation influenced the reduction of superficial peripapillary microvascular density. Linear regression analysis revealed that axial length was inversely correlated with the superficial peripapillary microvascular density. This finding may be partly attributable to the RNFL thinning induced by axial elongation. However, multivariate analysis revealed an independent association between axial length and average pRNFL thickness and superficial peripapillary microvascular reduction, which indicates that they had an independent effect on the superficial peripapillary microvasculature. The stretching of the microvascular network itself, secondary to the axial elongation in highly myopic eyes, might also contribute to the superficial peripapillary capillary loss.

In this study, we adopted axial length rather than dioptric values an explanatory value in multivariate regression model. Because the dioptric value can be affected by crystalline lens status and it is known that axial elongation is related to the mechanism of the myopic optic disc change. Previous studies demonstrated the significant correlation between axial length and myopic change such as peripapillary scleral thinning and optic disc change [21]. Liu *et al* [22] recently showed that axial length was more strongly associated with myopic retinal change than the refractive error.

Peripapillary choroidal circulation is closely related to the prelaminar and laminar blood supply [23]. Therefore, understanding the peripapillary choroidal circulation may provide important clues for the pathogenesis of glaucomatous optic neuropathy in myopia. However, direct visualization and quantification of choroidal vasculature using en-face OCT angiography poses several difficulties. First, the vascular flow in the choriocapillaris and choroidal microvasculature may occasionally be too slow, which would not generate a decorrelation signal and would preclude documentation of these small vessels on OCT angiography in some circumstances [24]. A small decorrelation signal usually serves as a source of unwanted image degradation. Signal attenuation, either by light absorption from retinal pigment epithelium (RPE) or by loss of coherency from multiple scattering, also contributes to the reduction of image quality. Second, projection artifacts of superficial vessels may lead to a false positive detection of vascularity within the choroidal layer. Third, segmentation errors are common in highly myopic eyes. To overcome these limitations, we restricted our study subjects to myopia with β-PPA. β-PPA is characterized by the absence of RPE and has few superficial retinal vessels within the area; therefore, we could minimize the possibility of signal attenuation and the effect of projection artifacts. In addition, all B-scan images were reviewed thoroughly and images with any segmentation errors were strictly excluded from the analysis.

In myopic eyes with β-PPA, longer axial length was not associated with the risk of deep peripapillary microvascular reduction. Considering that axial elongation causes the thinning of both RNFL and choroid, one might raise a question about the reason for the difference in influence of axial length on superficial and deep peripapillary microvasculature. We speculate that it may be due to the difference in regulatory capability of the vasculature to oxygen demand between retinal and choroidal circulations. Since choroidal circulation is mainly controlled by sympathetic innervations and is not autoregulated, choroidal vasculature might be less affected by the decreased oxygen demand compared with RPC [25]. Furthermore, as shown by histological analysis, β-PPA area is characterized by a closure of the choriocapillaris; thus, a substantial portion of deep peripapillary microvasculature measured in this study might be medium- or large-size choroidal vessels [26]. We hypothesized that larger vessels could be less vulnerable to the mechanical stretching secondary to axial elongation. This could also indicate that factors other than axial elongation might be involved in the deep peripapillary microvasculature.

**Fig 3:**
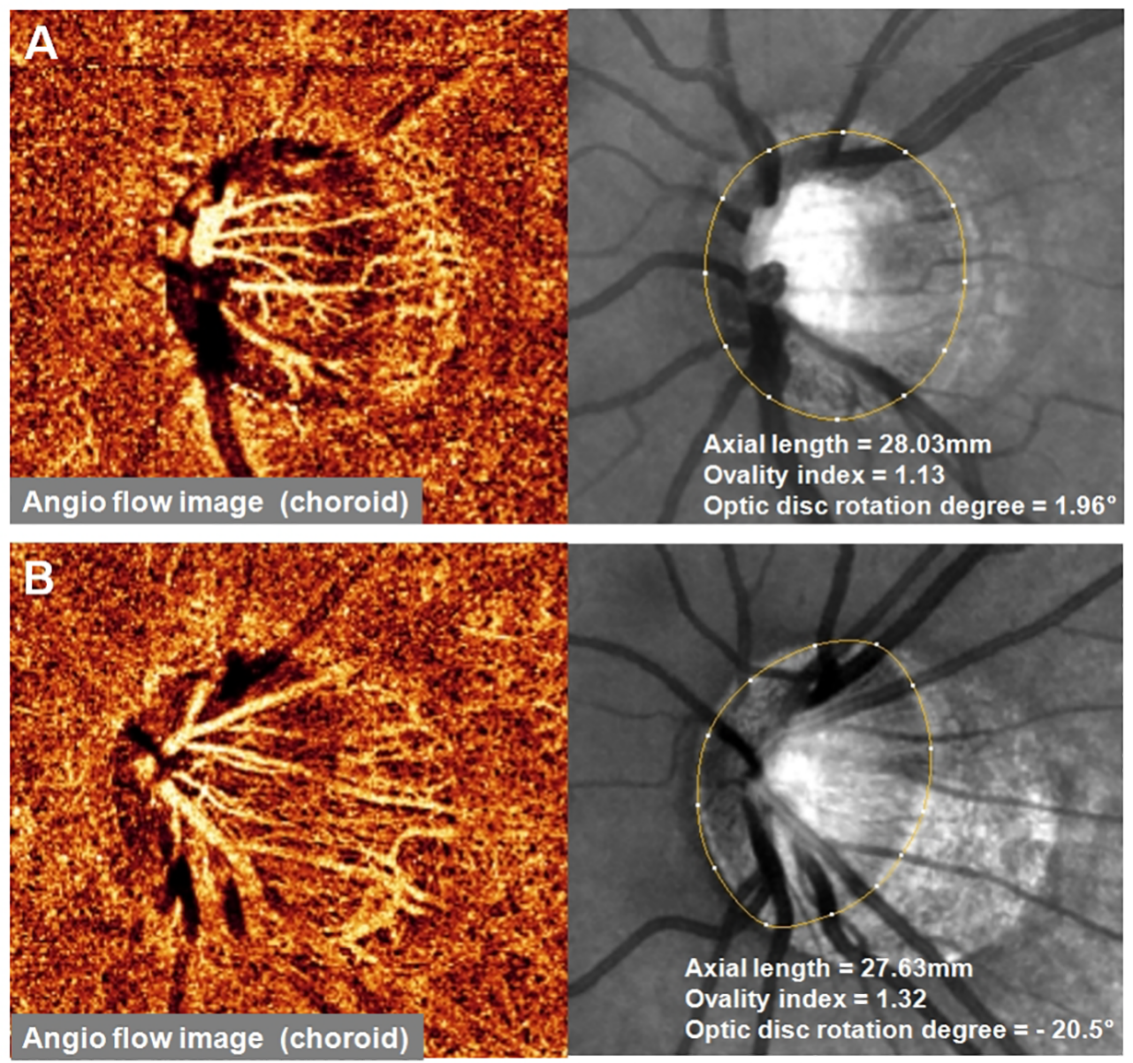
Two representative cases of healthy myopic eye with similar degree of axial myopia and different degrees of ovality index and optic disc rotation. (A) OCT angiography image of 22-year-old man. The signal strength of the image was 76 and the deep peripapillary microvascular density was 79.17%. (B) OCT angiography image of 28-year-old man. He had a larger ovality index and more inferiorly rotated optic disc. The signal strength of the image was 80 and the deep peripapillary microvascular density was 68.55%, which is a deep peripapillary microvascular reduction.

Interestingly, we observed that as the ovality index and the degree of inferior optic disc rotation increased, the risk of deep peripapillary microvascular reduction also increased. Fig 3 shows the representative two highly myopic eyes with similar degrees of axial myopia. They have considerably different deep peripapillary microvasculature densities in the β-PPA area depending on the optic disc morphology. Ovality index is a clinically useful finding that can be used to predict the presence of optic disc tilt [1, 27, 28]. Both optic disc tilt and rotation are characteristic morphologic changes found in a myopic optic disc and are thought to be the consequence of progressive stretching of the sclera during myopic shift. It could be speculated that these morphologic changes cause additional mechanical strain to microvasculature at the peripapillary region and result in deep peripapillary microvascular remodeling. Linear regression analysis revealed that the degree of inferior optic disc rotation was significantly correlated with the deep peripapillary microvascular density. We also found a significant difference in deep microvascular density between eyes with inferiorly and superiorly rotated optic discs.

The results of the current study may be one of the reasons why glaucoma patients with myopia have a higher prevalence of inferior rotation, and presumably, inferior rotation of the optic disc leads to a higher susceptibility for glaucomatous optic nerve damage, such as reduced pRNFL thickness, than superior rotation of the optic disc [3, 8]. A significant difference in the deep peripapillary microvascular density of the β-PPA area between the two groups suggests that a mechanical strain imposed in peripapillary sclera during the development of optic disc rotation might be different between the two groups, and this difference might affect the vulnerability to glaucomatous damage. Previously, Akagi *et al* [29] reported a microvascular dropout of the choriocapillaris in the PPA area in eyes with open angle glaucoma. Therefore, we hypothesize that direct mechanical damage to RNFL may not be the sole mechanism, and the alternation of peripapillary microvasculature, especially deep peripapillary microvascular density, might contribute to the development of glaucoma in myopic eyes with inferior rotation of the optic disc.

In multivariate analysis, we also found a significant association between the inside disc vessel density and the risk of deep peripapillary microvascular reduction. This may have been because the measurement of inside disc vessel density contains prelaminar tissue and lamina cribrosa. Since prelaminar tissue and lamina cribrosa are supplied by the choroidal circulation, this parameter reflects not only the superficial retinal vasculature at the optic disc but also the microvascular component at the peripapillary choroid.

Of note, linear regression analysis between the ovality index and deep peripapillary microvascular density was not statistically significant. Although the correlation of ovality index and optic disc tilt has been reported [1, 27], it is possible that ovality index is not a result of actual optic disc tilt measurement. Further investigation would be needed to clarify the association between optic disc tilt and peripapillary microvasculature in myopic eyes.

Our study shares the limitations associated with cross-sectional studies, which cannot confirm causation. Whether the vascular alterations in myopic eyes occur before or after the morphologic optic disc changes remains unclear. This issue would best be investigated in longitudinal studies. Second, this study was not performed on a general myopic population, and the inclusion criteria were confined to the myopic eyes having β-PPA. Thus, arbitrarily chosen cut-off values for the superficial and deep microvascular reduction in this study might not be applicable to general myopic population. Nevertheless, given that the β-PPA is important for the biomechanics of the ONH in glaucoma, the results from our study allow us to better understand glaucoma pathogenesis in myopic eyes. Third, we did not sub classify the PPA based on the presence of Bruch’s membrane. Specifically, it has been reported that PPA with intact Bruch’s membrane (β-zone PPA) is associated with glaucoma, whereas PPA devoid of Bruch’s membrane (γ-zone PPA) is unrelated to glaucoma [30, 31]. Finally, we cannot rule out the effect of noise on deep microvasculature measurements, which presumably is more apparent in highly myopic eyes. There may be a large component of noise in these measurements which could be related to image quality. However, to overcome this limitation, we strictly excluded the scan images with poor image qualities (signal strength index less than 70) from the analysis.

In conclusion, we have evaluated the factors associated with peripapillary microvascular reduction and found that the factors related to the microvascular density in the superficial and deep layers are different. Longer axial length was significantly associated with superficial microvascular reduction, whereas its influence on deep microvasculature was not significant. Larger ovality index and inferior rotation of the optic disc were associated with deep peripapillary microvascular reduction. Our findings may support that optic disc morphology and its vascular aspects may be relevant to glaucoma pathogenesis in myopic eyes.

## Supporting information

S1 Table (XLSX)
